# A hybrid digital parenting programme to prevent abuse of adolescents in Tanzania: statistical analysis plan for a pragmatic cluster randomised controlled trial

**DOI:** 10.1186/s13063-024-08292-6

**Published:** 2024-07-03

**Authors:** Jonathan Klapwijk, G. J. Melendez-Torres, Abigail Ornellas, Mwita Wambura, Angelique N. Chetty, Lauren Baerecke, Joyce Wamoyi, Lucie D. Cluver

**Affiliations:** 1 Department of Social Policy and Intervention, University of Oxford, Oxford, UK; 2 Faculty of Health and Life Sciences, University of Exeter, Exeter, UK; 3 Centre for Social Science Research, University of Cape Town, Cape Town, South Africa; 4 National Institute for Medical Research, Mwanza Research Centre, Mwanza, Tanzania; 5 Department of Psychiatry and Mental Health, University of Cape Town, Cape Town, South Africa

**Keywords:** Child abuse, Parenting, Adolescents, Digital, Low- and middle-income countries, Violence against children

## Abstract

**Background:**

Globally, violence against children poses substantial health and economic challenges, with estimated costs nearing USD 7 trillion. This prompts the urgent call for effective evidence-based interventions in preventing and mitigating violence against children. ParentApp is a mobile, open-source application designed to offer a remote version of the Parenting for Lifelong Health (PLH) programme. ParentApp is the first digital parenting intervention for caregivers of adolescents aged 10–17 years to be tested in low- and middle-income settings.

**Methods:**

This study is a pragmatic, two-arm, cluster-randomised trial in Mwanza, Tanzania’s urban and peri-urban areas. Assessments are set for baseline, 1 month post-intervention, and 12 months post-intervention. We randomised 80 clusters, each with about 30 caregiver-adolescent dyads, with a 1:1 ratio stratified by urban or peri-urban location. Both arms receive an entry-level smartphone preloaded with Kiswahili apps—ParentApp for intervention and WashApp control.

The primary method of analysis will be generalised linear mixed-effects models with adjustment for person-level characteristics and multiple imputation. In three-level models, measurement waves are nested within a person, nested within a sub-ward. Regressions will constrain groups to be equal at baseline and include covariates for stratification, percentage of male caregivers, and individual-level characteristics.

**Discussions:**

Preparations for the trial began in December 2022, including community mobilisation and sensitisation. Rolling recruitment, baseline data collection, and implementation onboarding took place between April and September 2023. One-month post-test data collection began in August 2023 and thus far achieved 97% and 94% retention rates for caregivers and adolescents respectively. Final post-test data collection will begin in September 2024, anticipated to run until April 2025. This SAP was submitted to the journal before the interim analysis to preserve scientific integrity under a superiority hypothesis testing framework.

**Trial registration:**

The trial was registered on the Open Science Framework on 14 March 2023: https://doi.org/10.17605/OSF.IO/T9FXZ.

The trial protocol was published in Trials 25, 119 (2024): Baerecke, L., Ornellas, A., Wamoyi, J. et al. A hybrid digital parenting programme to prevent abuse of adolescents in Tanzania: study protocol for a pragmatic cluster-randomised controlled trial. Trials 25, 119 (2024). https://doi.org/10.1186/s13063-023-07893-x.

## Introduction

### Background and rationale (7)

Violence against children is estimated to affect over a billion children a year [1]. Instances of violence adversely affect communities in low- and middle-income countries with the African continent experiencing one of the highest rates of verbal, physical, and sexual violence among children and adolescents [2, 3]. The repercussions of violence against children span multiple adverse and often long-term outcomes across the lifecycle and can lead to increased demands on health and welfare systems [4–6]. Global estimates of the cost of violence against children is nearly USD 7 trillion [7], resulting in an international commitment to ending violence against children as a strategic objective for sustainable development [8].

Parenting programmes have emerged as a cornerstone strategy in preventing and reducing violence against children, with evidence-based interventions demonstrating significant benefits in improving parenting practices and child outcomes [9]. However, the scale up of these programmes can be challenging and costly. In the wake of the COVID-19 pandemic, there is a global shift toward digital interventions through hybrid and remote mobile app design. The digital delivery of parenting programmes presents a viable solution to these challenges, potentially offering greater reach and accessibility in settings experiencing rapid increases in smartphone penetration [10]. Digital or hybrid digital interventions, which combine remote or in-person human support with digital delivery, may be a valuable delivery approach to increase the scale-up of parenting programmes at national levels. A systematic review identified 15 randomised trials of digital parenting programmes with promising results; however, these are concentrated in the Global North and target parenting of younger children. Few studies have looked at digital interventions for parenting adolescents in the Global South [11].

ParentApp for Teens (referred to hereafter as ParentApp) is a mobile application designed to offer a remote version of the Parenting for Lifelong Health (PLH) programme; PLH is an open-source, evidence-based intervention, tailored for families in LMICs, showing promise in reducing violence against children, enhancing positive parenting, and improving family financial management [12, 13]. ParentApp is the first digital parenting intervention for caregivers of adolescents aged 10–17 years to be tested in low- and middle-income settings. The app, designed specifically for contexts with limited to no internet, was developed between 2019 and 2022 through a seven-stage process that involved multi-phased co-development with various stakeholders, participatory engagement across 14 African countries, mixed-methods user testing, a feasibility pilot, a pre-post pilot, a cluster-randomised optimisation factorial trial, and two qualitative studies included in the feasibility pilot and optimisation trial. The adaptation and optimisation of ParentApp was conducted in partnership with the Universities of Oxford and Cape Town, IDEMS International, Tanzania’s National Institute for Medical Research (NIMR), Parenting for Lifelong Health, and Clowns Without Borders South Africa (CWBSA), with support from the Tanzania-based NGO Investing in Children and Strengthening their Societies (ICS) and extensive collaborations with researchers, programme specialists, technical experts, caregivers, and adolescents.

This trial aims to rigorously evaluate the effectiveness and cost-effectiveness of ParentApp in reducing maltreatment and sexual violence risks among adolescents aged 10–17 years through digital parenting programme delivery in urban and peri-urban communities. The trial is set in Mwanza, Tanzania, where the in-person PLH programme has been successfully delivered, and the scale-up of parenting programmes is a national commitment [14].

This paper presents the statistical analysis plan (SAP) of the ParentApp Randomised Controlled Trial, building upon the existing study protocol [15].

### Objectives (8)

The SAP is guided by the trial objectives, informing the choice of statistical methods, the handling of data, and the interpretation of results. The trial has the following objectives:

1. Determine the effectiveness of the ParentApp intervention by assessing if the intervention, compared to a control condition (WashApp), effectively decreases rates of adolescent maltreatment (physical and emotional abuse) and sexual violence victimisation and vulnerability at 1 month and 12 months post-intervention
2. Evaluate cost-effectiveness by analysing the economic viability of the ParentApp intervention by analysing its cost-effectiveness in achieving the primary and secondary outcomes, from a perspective that encompasses the public and health sector, and participant costs and benefits
3. Assess scalability and delivery by investigating the feasibility of delivering ParentApp at scale through local implementing partners, focusing on the programme’s accessibility and participant engagement within the Tanzanian context
4. Explore possible mechanisms of change through mediation and moderation analyses. To identify pathways through which ParentApp impacts behavioural outcomes, including changes in parenting practices, adolescent behaviour, and family dynamics

## Study methods

### Trial design (9)

The design of this study is a pragmatic, two-arm, cluster-randomised controlled trial (RCT) within the urban and peri-urban settings of Mwanza, Tanzania. The study’s assessments will be collected at baseline, followed by evaluations at 1 month post intervention and at 12 months post intervention. The trial encompasses the randomisation of 80 urban and peri-urban clusters, each consisting of approximately 30 caregiver-adolescent dyads. Randomisation will follow a structured design and be conducted at the cluster level, adhering to a strict 1:1 allocation ratio.

Caregivers in both the intervention arm and control arm will be provided with an entry level, locally sold, smartphone; phones will be preloaded with the Kiswahili version of ParentApp for the intervention arm and a simple hygiene programme, WASH App, for the control arm. The intervention group will also be added to facilitated WhatsApp groups for the exchange of insights and learnings among caregivers.

### Randomisation (10)

To increase community and participant acceptance of the outcome, a participatory randomisation approach will be used. This involves representatives from the selected communities drawing a number that corresponds to a concealed randomisation sequence. Clusters will be randomly assigned to either the control or intervention group with a 1:1 allocation in blocks of six stratified by urban vs peri-urban context. The implementing partner will notify the participating families of their allocation status after baseline data collection in their cluster is complete to ensure that participants are blind to allocation during the pre-test assessment. While participants will not be blind to their own treatment condition, the allocation status of participating families in other sites/clusters will be concealed, thus reducing the potential for contamination. Blinding will not be possible for facilitators and local research staff due to their involvement in programme implementation.

### Sample size (11)

The sample size is based on a detailed power calculation informed by preliminary pilot study data, using generalised Poisson linear mixed-effect models. These calculations posit that, to achieve a statistical power of 80% against an anticipated effect size of 0.95 and a control group mean outcome of 4, while accounting for potential attrition rates within both the control and intervention arms, a minimum ensemble of 80 clusters, each comprising 30 dyads, is required. The trial will therefore enrol a cohort of approximately 2400 caregiver-adolescent dyads or 4800 participants.

A subset of participants, comprising 40 to 60 caregivers and 20 to 30 adolescents, will be invited to engage in post-intervention qualitative interviews. These discussions are aimed at eliciting in-depth insights into the experiential dimensions of the programme, thereby enriching the quantitative data. Additionally, semi-structured interviews will be conducted with a representative sample of 10 implementing staff members, offering a comprehensive perspective on programme delivery dynamics.

Expanding the impact of the study, we intend to include adolescent siblings or co-resident adolescents present within approximately 50% of the participating households (estimated *n* = 2610) in the post-intervention assessment phase. This strategic inclusion aims to ascertain the broader familial impact of the intervention, thereby capturing any auxiliary effects on the household’s adolescent population not directly involved in the primary intervention activities.

### Framework (12)

Statistical analyses within this study will be conducted under the control of a superiority hypothesis testing framework. This approach has been selected to ascertain whether the intervention exhibits a statistically significant and clinically meaningful impact over the control condition.

### Statistical interim analyses and stopping guidance (13a, b, c)

Interim analyses will be conducted at 1 month post-intervention (4 months post-baseline). During the interim analysis, the nominal significance has been set at *p* < 0.001 to mitigate the risk of type I error or false-positive findings due to multiple testing across the trial’s timeframe. The residual nominal significance level for the final analyses will thus approximate *p* = 0.05. As such, while 95% confidence intervals will be calculated to facilitate the interpretation of the interim results, these results will not be used to make conclusive statements about the intervention’s effectiveness.

### Timing of final analysis (14)

Results will be analysed upon database lock, following completion of all data collection stages in all sites.

### Timing of outcome assessments (15)

Outcomes will be assessed at three time points: baseline, 1 month post-intervention (4 months post-baseline), and 12 months post-intervention (16 months post-baseline).

## Statistical principles

### Confidence intervals and p values (16, 17, 18)

All reported confidence intervals will be at the 95% level. Any hypothesis testing will be undertaken at the level of statistical significance corresponding to the prespecified alpha level of *α* = 0.05 with two-tailed tests for all analyses. Minimal clinically important differences are not well understood or established for many outcomes included in this analysis. Therefore, we will not explicitly benchmark findings against these differences in any primary analyses.

### Adherence and protocol deviations (19a, b, c, d)

Protocol deviations, if they occur, are likely to relate to the rollout and availability of the intervention. All protocol deviations will be systematically recoded and detailed in the final evaluation report of the trial. Adherence will be considered as part of the process evaluation which is not covered by this SAP.

### Analysis populations (20)

The analysis populations are defined at both the family and the cluster levels. In all cases, the analysis adheres to an intention-to-treat approach. This methodology ensures analysing participants strictly according to the randomisation allocation of their cluster. At the family level, the analysis will be based on the treatment allocation of the relevant cluster and include all dyads that meet the eligibility criteria, have provided informed consent, and have valid outcome data. Data are provided by one caregiver per family. Within families, we will identify one index child, defined as the child whose birthday is coming up next. If the child is aged between 10 and 17 years at baseline, that child will be eligible for entry in the adolescent sample. In an additional round of data collection for post hoc data analyses only (around 3–5 months after intervention), we will interview all other adolescent siblings (aged 10–17 at baseline, or who have turned 10 since baseline) or co-resident adolescents in households. Analysis methods for this cohort will be treated in an addendum to this statistical analysis plan.

## Trial population

### Screening data (21)

The principal investigators (PIs) and research managers will ensure comprehensive training on the trial protocol’s inclusion and exclusion criteria for all staff members and collaborating partners. This process will uphold the trial’s screening process integrity and ensure a consistent application of participant screening across the study. The PI or designate is responsible for final decisions regarding the eligibility of participants. In instances where screening outcomes render a potential participant ineligible to be included, the rationale for this exclusion will be clearly communicated to the participant. For the participants who are deemed ineligible, all relevant data collected during the screening assessment, including any informed consent documentation and records of completed study procedures, will be retained for the lifespan of the study. Participants who meet all eligibility criteria and complete informed consent procedures will be enrolled into the trial. All screening records for eligible participants will be included in the research record for that participant and household. To facilitate the screening process, a detailed screening log will be maintained and include the following information: the screening number, participant age, date of screening, the date informed consent was obtained, and the date of study enrolment. Participants who opt not to participate in the trial will be politely asked to provide reasons for their decision to decline. In these instances, it will be explicitly communicated that they are under no obligation to disclose their reasons and may decline without reason or comment. Declining participants will not be assigned a screening number but will be captured as refusals in the data logs. All responses will be recorded as part of the screening data. The screening log should be reviewed periodically by the PI or research managers to determine if there are any trends regarding ineligibility.

### Eligibility (22)

The selection of eligible communities will focus on urban and peri-urban sub wards in Mwanza, Tanzania. These communities must meet a specific eligibility criterion, specifically having at least 30 households that are qualified participants in the national Tanzania Social Action Fund (TASAF) programme. This requirement serves as a nationally recognised indicator of poverty and vulnerability, ensuring that the communities selected are those in genuine need. However, it is important to note that only a select group of these eligible households will be part of the programme during the trial period. The study will adopt a simplified version of the TASAF eligibility criteria, as outlined in the Interagency Social Protection Assessments [16] to facilitate the selection process. In addition, the selection will also prioritise households that do not currently own a smartphone.

The trial involves three distinct participant groups. This includes caregiver, adolescents, and the staff of the implementation partner. The selection of caregivers and adolescents will be confined to eligible clusters, which include sub wards within the Mwanza Region. Sub-wards are defined as the lowest administrative structure at the community level in urban settings in Tanzania.

For caregivers from the selected sub-wards to be eligible to participate in the trial, they are required to meet several inclusion criteria. Caregivers should be 18 years or older and be the primary caregiver of an adolescent aged between 10 and 17 years old. Additionally, the caregiver should cohabit with the adolescent, sharing the same household for a minimum of four nights per week over the preceding month. Basic literacy is a criterion, assessed through a simple screening question about their reading capabilities. Suitable responses include the ability to read with slight difficulty or ease. Caregivers who report illiteracy or have significant reading challenges will be excluded. Caregivers must also consent to engage with either the ParentApp (intervention) or WASH App (control) programme and provide written or oral informed consent for full study participation.

Adolescents eligible to be included in the study are required to be aged between 10 to 17 years old at the study’s outset. They need to be dependent on a caregiver who satisfies the above specified criteria for caregivers and who have provided written or oral assent. This should be over and above obtaining the required consent from the primary caregiver for full participation in the study.

Staff members from the implementing partners will qualify for the study if they are 18 years or older, have participated in the ParentApp facilitator training workshop, and can give written or oral informed consent for participation.

The study will exclude any potential participants who exhibit severe mental health issues or acute mental disabilities if these conditions impede their capacity to provide informed consent, adhering to ethical considerations.

### Recruitment (23)

The recruitment for this pragmatic cluster-randomised trial is designed to closely mirror the processes anticipated in a government-led scale-up, thereby ensuring the real-world applicability of our findings. Recruitment will work using a collaborative effort with established partnerships with UNICEF, the Tanzanian government, and the National Institute for Medical Research (NIMR), alongside local implementation by the Investing in Children and Strengthening their Societies (ICS) organisation.

Our recruitment strategy is multifaceted, aiming to engage caregiver-adolescent dyads through a variety of channels. These include leveraging existing community groups, such as farmer’s groups, that offer natural assemblies of our target demographic. In addition to this, we will employ both traditional and digital outreach recruitment methods.

Prior to the initiation of any recruitment activities, formal approval will be secured from local community and political leaders to ensure a collaborative recruitment approach. The community leaders will be asked to take an active role in the recruitment process, ensuring comprehensive community mapping and recruitment efforts. This process ensures that the recruitment procedure is not only respectful of local community structures but is also tailored to the unique socio-cultural context of each sub ward.

**Fig. 1:**
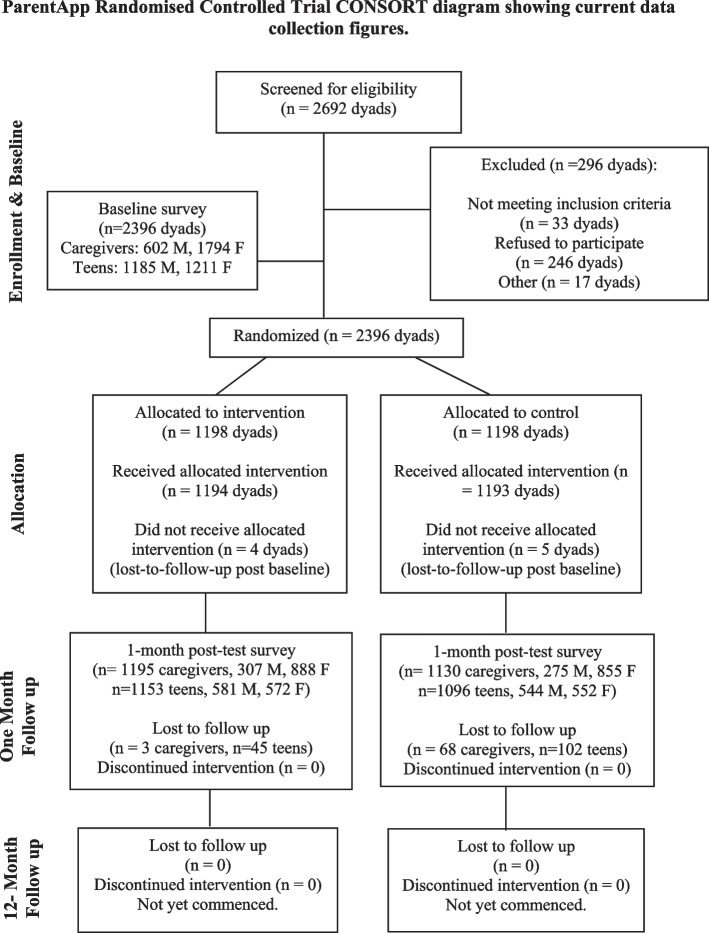
ParentApp Randomised Controlled Trial CONSORT diagram showing current data collection figures

Current recruitment and data collection figures are included in the CONSORT flow diagram (Fig. 1).

### Withdrawal/follow-up (24a, b, c)

Following the ethical principles guiding this trial, caregivers and adolescents have the explicit right to withdraw from the study at any point, without facing any type of repercussion or penalty. All collected data will be retained up to the point of withdrawal unless the participant requests otherwise. The study has a proactive monitoring mechanism designed to identify and address any adverse effects stemming from participation. Should it become evident that participation in the trial, or exposure to the intervention, has caused significant harm to participants or their families, the study team will take immediate action to suspend research activities. Types of harm that will be screened for include abuse, suicidality, intimate partner violence (IPV), and other forms of psychological or physical distress. Research activities will only resume if all issues can be adequately addressed, ensuring harm will not be repeated.

### Baseline patient characteristics (25a, b)

Participant recruitment and baseline assessment are undertaken on a rolling basis. Randomisation will occur once complete strata have been defined, ensuring the allocation of treatment groups is as unbiased as possible. No caregiver baseline data are used at the point of randomisation. Blocks of clusters within strata will be randomised to account for potential population differences between regions.

For each cluster, we will report on the following key characteristics:

(i) District: The specific administrative district within the region of Mwanza
(ii) Ward: The ward within the district, providing a finer geographical categorisation
(iii) Cluster: A further breakdown within the ward to identify specific participant groups

Baseline characteristics collected from caregivers include:

(i) Age
(ii) Gender
(iii) Relationship with adolescent
(iv) Reading proficiency

Adolescents’ baseline characteristics, as reported by their caregivers, include:

(i) Age
(ii) Gender

## Statistical analysis

### Outcome definitions (26a, b, c)

This trial explores several outcomes, including primary and secondary, socio-demographic measures, cost-effectiveness, implementation, and exploratory. As previously noted, data collection will occur at three timepoints: baseline, 1 month post-intervention, and again at 12 months post-intervention. To ensure consistent reliability and validity across the study’s scope, the outcome measurements will be collected from both caregivers and adolescents and, unless specified otherwise, be reported independently. A consistent recall period of ‘the past 4 weeks’ will be used to optimise the precision of potential behaviour change detection attributable to the intervention. All outcomes are supported by an established theoretical framework, with intervention effects rigorously evaluated through empirical analysis. Tools will be back translated into Kiswahili and undergo extensive local review to ensure language accuracy and cultural relevance; certain measures have been adapted following feedback from the pilot phase of the trial with the aim of enhancing comprehensibility and contextual relevance.

#### Primary outcomes

Primary outcomes for both caregivers and adolescents include child maltreatment, sexual violence victimisation, and sexual violence vulnerability. *Child maltreatment* includes both physical [1a] and emotional [1b] abuse and will be assessed using subscales of the Caregiver and Child Versions of the International Society for the Prevention of Child Abuse and Neglect Screen Tool for Trials (ICAST-Trials) [17].

*Sexual violence victimisation [3a; 3b; 3c; 3h]* will combine measures and items of contact sexual abuse, non-contact sexual abuse, alcohol-facilitated forced sex, and transactional sex; these measures are from adolescent reports only. *Sexual violence vulnerability [3e]*, both adolescent and caregiver reported*,* will combine items of exposure to high-risk situations for sexual violence. These two outcomes will be evaluated using a combination of items from the ICAST-Trial measures [17], the CDC Violence Against Children and Youth Surveys [18], and a locally derived sexual violence vulnerability scale developed through consultation with families and practitioners. This approach differs slightly from what was reported in the study protocol, in that these two outcomes will then be combined to form a single primary outcome of *sexual violence victimisation and vulnerability [3a; 3b; 3c; 3h; 3e]*. To address the intricate dynamics of sexual violence victimisation and vulnerability, additional exploratory analysis will disaggregate outcomes by gender.

#### Secondary outcomes

The secondary outcomes are presented in Table 2.

#### Exploratory outcomes

- *Use of internet and exposure to online violence risk*—the Global Partnership to End Violence Against Children’s Disrupting Harm project [19] (adapted)
- *Digital literacy*—questions developed by the research team to assess digital skills
- *Adolescent externalising and internalising problem behaviour*—the Child and Adolescent Behavior Inventory (CABI) [20]. Items are caregiver reported only
- *Adolescent internalising mental health distress, depression and anxiety – PHQ (adolescent only)*
- *Substance use*—the WHO Alcohol Use Disorders Identification Test [21] and the WHO Global School-based Health Survey [22] (adapted)
- *Engaged responsive parenting* encompassing cross-cultural indicators of playful learning [23], characteristics of learning through play [24, 25] and positive parenting—the APQ positive parenting and involvement subscales, the parent-child communication scale, and parent support for school. Caregiver’s engagement with playful activities in the app (e.g. number of playful activities completed) will be measured through app engagement data

#### Socio-demographic measures

Baseline socio-demographics for both caregiver and adolescent will include items from the Multiple Indicator Cluster Surveys (MICS) [26] as well as general questions:

- Age
- Gender
- Assessment of disability (baseline and 1 month follow-up)
- Assessment of basic literacy
- Household structure
- Adolescent’s relationship to the caregiver
- Household employment
- School enrolment
- Relationship status
- Orphanhood
- Access to social protection (e.g. government cash transfers)

#### Cost-effectiveness outcomes

Implementation costs will be calculated through weekly surveys completed by facilitators, recording time and data spent in WhatsApp group live chats, additional remote support, any preparatory efforts from facilitators, and basic participant engagement statistics. This will enable accurate and real-time data to inform implementation costs. Delivery costs will be retrospectively calculated, including facilitator training and support, mobile device provision, programme onboarding, and app maintenance. For cost-effectiveness measurements, a visual analogue 0–100 scale will be used to inform quality-adjusted life years (QALY) in the pre and post surveys for both adolescents and caregivers. To better estimate cost and savings of the intervention across multiple outcomes and VAC pathways, we will deploy a multi-outcome discounted cost-effectiveness approach [27, 28].

#### Implementation outcomes

Implementation outcomes will be assessed to understand intervention fidelity, uptake, and retention. Data on the amount, frequency, and duration of app usage, as well as the nature, scope, and depth of content engagement, will be collected through app-generated statistics. Weekly facilitator surveys, as described under cost-effectiveness outcomes, will be used to collect facilitator-reported experiences such as implementation challenges, successes, and other relevant insights. To explore the quality of support, a random sampling of the moderated WhatsApp live chat sessions will be assessed; the team will ensure complete transparency in any monitoring activities and participant confidentiality will be maintained. Interviews with select implementing staff will provide supplementary qualitative insights.

Implementation outcomes will further include exploration of intervention acceptability, benefits, and challenges through qualitative interviews with caregivers, adolescents, and implementing staff. Open-ended questions on ParentApp acceptability, cultural relevance, delivery experiences, usage satisfaction and engagement, and perceived benefits and challenges will be explored. Focus group discussions with implementing staff will be explore facilitator experiences of intervention delivery, processes, barriers, and recommendations for future implementation.

#### Exploratory interviews with siblings

With a small amount of additional funding, exploratory data collection will take place with adolescent siblings or co-residents in participating households. The survey tool will align with the 1 month follow-up survey administered to trial caregiver-adolescent dyads, with minor alterations. This additional exploratory outcome will allow us to ascertain potential spill-over effects of the intervention to other children within the household.

### Analysis methods (27a, b, c, d, e)

#### Analysis methods for primary and secondary outcomes

The primary method of analysis will be generalised linear mixed-effects models to account for the hierarchical nature of the data. This will involve specifying a three-level model with adjustment for person-level characteristics and multiple imputation. In this three-level model, measurement waves are nested within a person, and this is nested within a sub-ward (i.e. cluster). Each regression will delineate the relationships at each level where necessary. At the cluster level (level 3), the model incorporates terms for randomisation stratification variables and contextual covariates such as the percentage of male caregivers within each cluster. At variance with the protocol, a term for intervention allocation will not be included due to the repeated measures nature of the analysis (and the subsequent uninterpretability of a ‘baseline’ intervention term). This is equivalent to constraining the intercepts to be equal by arm. At the individual level (level 2), the model adjusts for demographic characteristics centred at the overall sample mean. For caregiver report models, this includes caregiver age, caregiver gender, caregiver-adolescent relationship, caregiver disability, child age, child gender, and child disability; caregiver gender specifically is centred at the overall cluster mean to facilitate interpretable comparisons. For child report models, this includes caregiver-adolescent relationship, child age, child gender, and child disability. At level 1, the model includes a categorical time variable and interactions between intervention allocation and time (i.e. the test of intervention effectiveness). If an event occurs whereby more than participant (adolescent or caregiver) is recruited from a single household, we will implement a clustered standard error structure on level 2. If a three-level model fails to converge, we will use a two-level model with measurement waves within person, clustering standard errors by sub-ward.

The regression link functions will be tailored based on the distributional characteristic of each outcome measure. For example, a logit link will be used when outcome measures are binary and a Poisson link for outcome measures with count data which include integer values from 0.

The intervention’s effectiveness will be evaluated through multiparameter Wald tests or likelihood ratio tests as appropriate, comparing models with and without intervention-by-time interactions to assess the totality of the intervention’s impact. Furthermore, the use of Wald tests will assess the significance of specific fixed effects model coefficients, particularly in relation to the intervention-by-time interactions. In addition to the primary analysis, we will conduct both adjusted analyses with unimputed data and unadjusted analyses with unimputed data, which include terms for stratification, time, and intervention-by-time, to provide a baseline estimate of intervention effectiveness without covariate adjustments.

Both Wald tests and marginal effects plots will be used to evaluate differences of outcome interactions by intervention group and will be supported by the computation of intra-cluster correlation coefficient (ICC) for hierarchical data. Depending on the distribution of the data, i.e. binary or Poisson, median incidence rate ratios (IRR) or odds ratios for hierarchical and non-normally distributed data will be respectively calculated.

The *p*-value threshold will be 0.05 for all pre-specified analyses. However, due to multiple comparisons planned for exploratory analyses, a sharpened *q*-value will be used with respect to all exploratory analyses to adjust the *p*-value threshold for statistical significance to reduce the likelihood of false positives.

#### Quality assurance

As a minimum, derivation of each of the primary outcomes will be undertaken twice, independently, with primary analyses also performed twice, independently. Dependent on capacity, derivation of key secondary outcomes will also be undertaken twice, independently where possible.

### Missing data (28)

To account for the hierarchical structure of the data (with individuals nested within families, and families within clusters), we will employ a two-level ‘wide’ multiple imputation strategy utilising fully conditional specifications. This approach allows us to impute missing data by specifying a model that includes intervention effects and stratifiers at the cluster level (level 2), along with the relevant baseline person-level predictors at the individual level (level 1). This approach requires ignoring possible clustering of standard errors. An example is where more than one caregiver is recruited in a household, therefore generating biased estimates of ICCs. As the substantive variance partition of interest is at level three, the impact of this would likely be negligible. A consistent imputation rule will be used to determine where scales are imputed, items are imputed, or another strategy is used.

### Additional analyses (29)

#### Gender-disaggregated analyses

Specific outcomes described in Tables 1, 2, and 3 will be analysed disaggregated by caregiver or adolescent gender. These will be estimated as unadjusted models including terms for stratification, time, and intervention-by-time only.

**Table 1:** Primary outcomes

Outcome	Informant	Gender disaggregated	Measure
Child maltreatment includes both physical and emotional abuse	Both	No	Caregiver and Child Versions of the International Society for the Prevention of Child Abuse and Neglect Screen Tool for Trials (ICAST-Trials) [17] (CG Range 0–140; AD Range 0–140)
Child physical abuse	Both	No	ICAST-Trial Caregiver and Child Versions Physical Abuse subscale [17] (CG Range 0–40; AD Range 0–40)
Child emotional abuse	Both	No	ICAST-Trial Caregiver and Child Versions Emotional Abuse subscale [17] (CG Range 0–100; AD Range 0–100)
Sexual violence victimisation and vulnerability	Both	Yes (adolescent)	Combining items of the ICAST-Trial Caregiver and Child Versions of Contact and Non-Contact Sexual Abuse subscale, Alcohol-Facilitated Forced Sex subscale [17], the WHO’s Violence Against Women Instrument and Global Partnership to End Violence Against Children’s Disrupting Harm Project Transactional Sex items. The CDC Violence Against Children and Youth Surveys [18], and a locally derived sexual violence vulnerability scale developed through consultation with families and practitioners (CG Range 0–90; AD Range 0–180)
Sexual violence victimisation	Adolescent	Yes (adolescent)	ICAST-Trial Caregiver and Child Versions of Contact and Non-Contact Sexual Abuse subscale, Alcohol-Facilitated Forced Sex [17], the WHO’s Violence Against Women Instrument Transactional Sex item and Global Partnership to End Violence Against Children’s Disrupting Harm project instrument (AD Range 0–90)
Sexual violence vulnerability	Both	Yes (adolescent)	CDC Violence Against Children and Youth Surveys [18], and a locally derived sexual violence vulnerability scale developed through consultation with families and practitioners (CG Range 0–90; AD Range 0–90)

**Table 2:** Secondary outcomes

Outcome	Informant	Gender disaggregated	Measure
Parental supervision, inconsistent discipline, positive parenting, positive involved parenting	Both	No	Alabama Parenting Questionnaire subscales [29] (CG Range 0–310; AD Range 0–310)
Child neglect	Both	No	ICAST-Trial Caregiver and Child Versions Neglect subscale [17] (adapted for low-income contexts where neglect may be unintentional/poverty-related) (CG Range 0–30; AD Range 0–60)
Caregiver and adolescent attitudes to physical punishment	Caregiver	No	UNICEF Multiple Indicator Cluster Surveys (MICS) 5 Child Discipline module [26] (CG Range 0–10)
Sexual violence victimisation risk planning	Both	No	Scale developed for a PLH RCT in South Africa [12] (single item) (CG Range 0–10; AD Range 0–10)
Intimate partner violence (IPV) experience, witnessing (adolescent report only), and perpetration (caregiver report only)	Both	Yes (except for witnessing)	WHO’s Violence Against Women Instrument (VAWI) [30] (adapted) ICAST-Trial Child Version [17] (select items) (CG Range 0–100; AD Range 0–120)
Gender equitable behaviours (caregiver report only)	Caregiver	Yes (caregiver)	Survey developed by researchers at the London School of Hygiene and Tropical Medicine (LSHTM); used in an RCT of a violence prevention intervention in Tanzania [31]. Survey developed by researchers of an RCT of a gender-transformative violence prevention intervention in Rwanda [32] (CG Range 0–40)
Caregiver and adolescent attitudes towards gender roles	Both	Yes (informant)	Attitudes toward gender roles section of the WHO Multi-Country Study on Domestic Violence [30] (CG Range 0–60; AD Range 0–60)
Caregiver support for school	Caregiver	No	Parent involvement and support of education [33] (CG Range 0–50)
Parent communication	Caregiver	No	Fast Track Intervention Study’s Parent-Child Communication Scale [34] (adapted) (CG Range 0–60)
Economic hardship	Both	No	Monthly shortfalls of basic necessities, such as clothes, soap, and school equipment (CG Range 0–8; AD Range 0–80)
Financial self-efficacy [9h] and family financial management	Both	No	Items on borrowing (from loan sharks and others), saving, and budgeting (CG Range 0–30; AD Range 0–30)
Parenting stress	Caregiver	No	Parental Stress Scale [35] (CG Range 0–70)
Mental health distress	Both	No	Patient Health Questionnaire 4 (PHQ-4) [36] (CG Range 0–40; AD Range 0–40)
Social support	Both	No	Medical Outcomes Study Social Support Survey [37] (CG Range 0–80; AD Range 0–80)
Sexual risk behaviour	Adolescent	Yes (adolescent)	CDC Violence Against Children and Youth Surveys (VACS) [18]. South African Demographic and Health Survey [38]. One item on age-disparate sex (AD Range 0–30)

**Table 3:** Exploratory outcomes

Outcome	Informant	Gender disaggregated	Measure
Use of internet and exposure to online violence risk	Both	No	The Global Partnership to End Violence Against Children’s Disrupting Harm project [19] (adapted) (CG Range 0–20; AD range 0–110)
Digital literacy	Both	No	Questions developed by the research team to assess digital skills (CG Range 0–40; AD Range 0–40)
Adolescent externalising and internalising behaviour	Caregiver	No	The Child and Adolescent Behavior Inventory (CABI) [20] (CG Range 0–140)
Substance use	Both	No	WHO Alcohol Use Disorders Identification Test [21] and the WHO Global School-based Health Survey [22] (adapted) (CG Range 0–20; AD Range 0–20)
Engaged responsive parenting	Both	No	Encompassing cross-cultural indicators of playful learning [23], characteristics of learning through play [24, 25] and positive parenting. Measures include the APQ positive parenting and involvement subscales, the parent-child communication scale, and parent support for school. Caregiver’s engagement with playful activities in the app (e.g. number of playful activities completed) will be measured through app engagement data (CG Range 0–260; AD Range 0–150)

#### Methods for additional analyses

The trial extends the analysis to investigate the effects of the intervention by mediation, moderation and community spread. The goal of these additional analyses is to explore the pathways through which the intervention impacts specific outcomes, to understand the conditions where these effects are amplified or attenuated, and how the intervention’s impact spreads within the community.

Mediation analysis will be conducted in instances where a significant effect of the intervention on both a potential mediator at post-intervention and on a primary outcome at the 12-month follow-up stage is observed. Utilising a two-level model that include individuals nested within clusters, our approach will incorporate a 2-1-1 mediation framework [39]. The mediation model will explore the interactions between the intervention allocation and the cluster-level mean of the mediator, together with outcome’s cluster-level random intercept at the 12-month follow-up, adjusting for baseline characteristics. The differentiation of pathways between the mediator and outcome, at both the individual and cluster level, will allow us to identify any contextual mediating effects.

The aim of the moderation analysis is to explore differential effects of the intervention, considering both the individual-level and cluster-level characteristics. The analysis will mirror the vector of level 2 characteristics used in the main outcome models. By centring the moderators within their context and examining the interactions at both levels within our hierarchical data structure, we will be able to identify variations in the intervention’s efficacy across different groups and contexts. Where feasible with data structures, moderated mediation hypothesis testing will be conducted using a multiple-group multilevel structural equation modelling approach [40]. Using level 3 variables as stratifiers, we will rerun mediation models to assess whether pathways comprising indirect effects are different over strata. The final analytic plans for mediation and moderation analyses will be submitted to Open Science Framework a priori to conducting these analyses.

Finally, we will examine community spread using standard indicators of intervention exposure in follow-up surveys. We will report descriptive statistics characterising the extent of intervention exposure in control clusters. If appropriate, we will use a multilevel complier average causal effect analysis to recover the ‘true’ effect of exposure to the intervention, focusing on primary outcomes and considering intervention exposure for each timepoint.

### Harms (30)

Aligning with the highest ethical standards and the universal principles of human research ethics, with respect for person, beneficence, and justice, this trial is designed to safeguard all participants through every phase of the trial. Ethical principles will be strictly enforced during all screening, recruitment, onboarding, data collection, and intervention engagement stages. Understanding the sensitive nature of the subject matter that is dealt with during the trial, there exists the potential that participants may disclose experiences of ongoing violent practices, whether they are the recipient or perpetrator of such acts towards themselves, their children, and/or partners. In line with this, the informed consent process ensures clarity and transparency regarding the handling of highly sensitive information, especially that which pertains to harmful practices. The participants are informed that, if necessary, certain information may need to be disclosed if the participant and/or members of their family are at risk of harm. To ensure that all members of the research team are committed to the ethical guidelines, all research assistants and implementation facilitators will undergo training on how to identify and respond to signs of harm. Should it be determined that the participation or involvement in the trial has led to significant harm for the participant and/or their family (including, but not limited to, abuse, suicidality, intimate partner violence, or other severe psychological or physical harm), the research team will cease all further activities until the issue can be resolved and ensure it is not repeated.

### Statistical software (31)

Quantitative data will be cleaned and analysed in R, R studio, and Stata v18.0.

## Trial status and discussion

Preparations for the trial began in December 2022, including community mobilisation and sensitisation. Due to a Marburg outbreak in the region, recruitment activities were put on hold mid-January 2023 and resumed once the outbreak had been declared safely contained in late March 2023. Rolling recruitment, baseline data collection, and implementation onboarding took place between April and September 2023. The final groups completed the 14-week programme in December 2023. One-month post-test data collection began in August 2023, and by March 2024, we had achieved 97% and 94% retention rates for caregivers and adolescents respectively. The exploratory sibling study commenced in April 2024 and is ongoing. Final post-test data collection (12-month follow-up) will begin in September 2024, and we anticipate will run until April 2025. This SAP was submitted to the journal before the interim analysis to preserve scientific integrity under a superiority hypothesis testing framework. The statistician has liaised with the data management team to ensure that any documents which could affect the statistics in the trial are referenced in this SAP. Nonstandard statistical methods were not used.

## Data Availability

Data sharing is not applicable to this article as no datasets were generated or analysed. Some datasets generated by the study will be made available via an open-access repository once fully anonymised and after the main study findings have been published.

## Abbreviations

CABI: Child and Adolescent Behaviour Inventory
CDC: Centres for Disease Control and Prevention
COREQ: Consolidated Criteria for Reporting Qualitative Research
CWBSA: Clowns Without Borders South Africa
GDPR: General Data Protection Regulation
GPI: Global Parenting Initiative
PLH: Parenting for Lifelong Health
ICAST: International Society for the Prevention of Child Abuse and Neglect Screening Tool
ICS: Investing in Children and Strengthening their Societies
IDEMS: Innovations in Development, Education and the Mathematical Sciences
INNODEMS: Innovations in Development, Education and the Mathematical Sciences
IPV: Intimate partner violence
LSHTM: London School of Hygiene and Tropical Medicine
MICS: Multiple Indicator Cluster Survey
MSI: Management Systems International
M&E: Monitoring and Evaluation
NGO: Non-governmental organisation
NIMR: National Institute for Medical Research (Tanzania)
ODK: Open Data Kit
PLH: Parenting for Lifelong Health
PHQ: Patient Health Questionnaire
SIM: Subscriber Identity Module
TASAF: Tanzania Social Action Fund
TSC: Trial steering committee
UNICEF: United Nations Children’s Fund
VAWI: Violence Against Women Instrument
WASH: Water, Sanitation and Hygiene
WHO: World Health Organization

## References

[1] Hillis S, Mercy J, Amobi A, Kress H (2016). Global prevalence of past-year violence against children: a systematic review and minimum estimates. Pediatrics..

[2] African Partnership to End Violence Against Children (APEVAC) and African Child Policy Forum (ACPF). Violence against children in Africa: a report on progress and challenges. Addis Ababa: ACPF. 2021. https://violenceagainstchildren.un.org/sites/violenceagainstchildren.un.org/files/2021/violence_against_children_in_africa_a_report_on_progress_and_challenges.pdfAccessed 16 May 2022.

[3] African Child Policy Forum. Sexual exploitation of children in Africa-a silent emergency. African Child Policy Forum; 2019. http://www.ci.uct.ac.za/sexual-violence/reports/sexual-exploitation-of-children-in-africa-a-silent-emergency. Accessed 21 Nov 2022.

[4] Hughes K, Bellis MA, Hardcastle KA, Sethi D, Butchart A, Mikton C (2017). The effect of multiple adverse childhood experiences on health: a systematic review and meta-analysis. Lancet Public Health..

[5] Norman RE, Byambaa M, De R, Butchart A, Scott J, Vos T (2012). The long-term health consequences of child physical abuse, emotional abuse, and neglect: a systematic review and meta-analysis. PLOS Med..

[6] Thornberry TP, Henry KL (2013). Intergenerational continuity in maltreatment. J Abnorm Child Psychol..

[7] UNICEF. Violence against children. 2020. https://www.unicef.org/protection/violence-against-childrenAccessed 21 Nov 2022.

[8] UNICEF. Global Annual Results Report 2021: Goal Area 3: every child is protected from violence and exploitation. New York, NY: UNICEF; 2022. https://www.unicef.org/media/121671/file/%20Global-annual-results-report-2021-goal-area-3.pdf. Accessed 16 May 2022.

[9] World Health Organization. WHO guidelines on parenting interventions to prevent maltreatment and enhance parent-child relationships with children aged 0–17 years. World Health Organisation. 2022. https://www.who.int/news/item/15-09-2022-guideline-on-parent-training-to-prevent-child-maltreatment-and-promote-the-positive-development-of-children-aged-0-17-yearsAccessed 21 Nov 2022. 36810030

[10] GSMA. (2021). The Mobile Economy Sub-Saharan Africa. https://www.gsma.com/mobileeconomy/wp-content/uploads/2021/09/GSMA_ME_SSA_2021_English_Web_Singles.pdf.

[11] Florean IS, Dobrean A, Păsărelu CR, Georgescu RD, Milea I (2020). The efficacy of internet-based parenting programs for children and adolescents with behavior problems: a meta-analysis of randomized clinical trials. Clin Child Fam Psychol Rev..

[12] Cluver LD, Meinck F, Steinert JI, Shenderovich Y, Doubt J, Romero RH (2018). Parenting for Lifelong Health: a pragmatic cluster randomised controlled trial of a non-commercialised parenting programme for adolescents and their families in South Africa. BMJ Glob Health..

[13] Lachman JM, Alampay LP, Jocson RM, Alinea C, Madrid B, Ward C (2021). Effectiveness of a parenting programme to reduce violence in a cash transfer system in the Philippines: RCT with follow-up. Lancet Reg Health West Pac..

[14] Ministry of Health, Community development, gender, elderly and children. National Plan of Action to End Violence Against Women and Children in Tanzania 2017/18–2021/22. 2016. https://www.unicef.org/tanzania/reports/national-plan-action-end-violence-against-women-and-children-tanzania-20178-20212Accessed 22 Nov 2022.

[15] Baerecke L, Ornellas A, Wamoyi J (2024). A hybrid digital parenting programme to prevent abuse of adolescents in Tanzania: study protocol for a pragmatic cluster-randomised controlled trial. Trials.

[16] TASAF, EU Social Protection Systems Programme and International Labour Organization. Assessment of TASAF PSSN in Tanzania using the ISPA-PWP Tool. 2017. https://www.sdgfund.org/publication/assessment-tasaf-pssn-tanzania-using-ispa-pwp-tool. Accessed 16 May 2023.

[17] Meinck F, Boyes ME, Cluver L, Ward CL, Schmidt P, DeStone S (2018). Adaptation and psychometric properties of the ISPCAN Child Abuse Screening Tool for use in trials (ICAST-Trial) among South African adolescents and their primary caregivers. Child Abuse Negl..

[18] Nguyen KH, Kress H, Villaveces A, Massetti GM (2019). Sampling design and methodology of the Violence Against Children and Youth Surveys. Inj Prev J Int Soc Child Adolesc Inj Prev..

[19] ECPAT, INTERPOL, UNICEF. Disrupting Harm in Tanzania: evidence on online child sexual exploitation and abuse. Global Partnership to End Violence against Children; 2022. https://www.unicef.org/innocenti/media/4131/file/DH-Tanzania-Report-2022.pdf. Accessed 26 May 2023.

[20] Cianchetti C, Pasculli M, Pittau A, Campus MG, Carta V, Littarru R (2017). Child and Adolescent Behavior Inventory (CABI): standardization for age 6–17 years and first clinical application. Clin Pract Epidemiol Ment Health CP EMH..

[21] Test Saunders JB, Aasland OG, Babor TF, de la Fuente JR, Grant M (1993). Development of the Alcohol Use Disorders Identification Test (AUDIT): WHO collaborative project on early detection of persons with harmful alcohol consumption II. Addiction.

[22] World Health Organization. Global School-based Student Health Survey (GSHS). 2018. Available from: https://www.who.int/teams/noncommunicable-diseases/surveillance/systems-tools/global-school-based-student-health-survey.

[23] Mardell B, Ryan J, Krechevsky M, Baker M, Schulz S, Constant YL (2023). A pedagogy of play: supporting playful learning in classrooms and schools.

[24] Jukes M, Betts K, Dubeck MP, Edwards L, Nduku T, Staskowicz E, et al. The LEGO Foundation Playful Learning Across the Years (PLAY) measurement toolkit: full report. The LEGO Foundation. 2022. https://cms.learningthroughplay.com/media/nlvm4lve/play-toolkit-development-technical-report-1.pdfAccessed 26 May 2023.

[25] Hirsh-Pasek K, Zosh JM, Golinkoff RM, Gray JH, Robb MB, Kaufman J (2015). Put-ting education in ‘educational’ apps: lessons from the science of learning. Psychol Sci Public Interest J Am Psychol Soc..

[26] The United Nations Children’s Fund: Division of Policy and Planning. Multiple indicator cluster survey manual, 2005: Monitoring the situation of children and women. New York: UNICEF; 2006. https://mics.unicef.org/files?job=W1siZiIsIjIwMTUvMDQvMDIvMDYvMzcvMDYvMTE5L011bHRpcGxlX0luZGljYXRvcl9DbHVzdGVyX1N1cnZleV9NYW51YWxfMjAw NS5wZGYiXV0&sha=dd2e54d1ddd61cdbAccessed 26 May 2022.

[27] McCaffrey N, Agar M, Harlum J, Karnon J, Currow D, Eckermann S (2015). Better informing decision making with multiple outcomes cost-effectiveness analysis under uncertainty in cost-disutility space. PLoS ONE..

[28] Walker S, Griffin S, Asaria M, Tsuchiya A, Sculpher M (2019). Striving for a societal perspective: a framework for economic evaluations when costs and effects fall on multiple sectors and decision makers. Appl Health Econ Health Policy..

[29] Essau CA, Sasagawa S, Frick PJ (2006). Psychometric properties of the Alabama parenting questionnaire. J Child Fam Stud..

[30] García-Moreno C, Jansen HAFM, Ellsberg M, Heise L, Watts C (2005). WHO multi-country study on women’s health and domestic violence against women: initial results on prevalence, health outcomes and women’s responses.

[31] Abramsky T, Kapinga I, Mshana G, Lees S, Hansen CH, Hashim R (2020). Couples data from north-western Tanzania: insights from a survey of male partners of women enrolled in the MAISHA cluster randomized trial of an intimate partner violence prevention intervention. PLoS ONE..

[32] Doyle K, Levtov RG, Barker G, Bastian GG, Bingenheimer JB, Kazimbaya S (2018). Gender-transformative Bandebereho couples’ intervention to promote male engagement in reproductive and maternal health and violence prevention in Rwanda: findings from a randomized controlled trial. PLoS ONE..

[33] McCarty CM, Doyle SR. Parent Questionnaire (Grade 4+) (Fast Track Project Technical Report Addendum). 2001. https://fasttrackproject.org/. Accessed 01 Aug 2022.

[34] Ceballo R, Maurizi LK, Suarez GA, Aretakis MT (2014). Gift and sacrifice: parental involvement in Latino adolescents’ education. Cultur Divers Ethnic Minor Psychol..

[35] Berry JO, Jones WH (1995). The Parental Stress Scale: initial psychometric evidence. J Soc Pers Relatsh..

[36] Stanhope J (2016). Patient Health Questionnaire-4. Occup Med..

[37] Sherbourne CD, Stewart AL (1982). The MOS social support survey. Soc Sci Med..

[38] SADHS. South Africa Demographic and Health Survey 2016. National Department of Health; 2019. https://dhsprogram.com/pubs/pdf/FR337/FR337.pdf. Accessed 22 May 2022.

[39] Little RJ, Yau LHY (1998). Statistical techniques for analyzing data from prevention trials: treatment of no-shows using Rubin’s causal model. Psychol Methods..

[40] Melendez-Torres GJ, Warren E, Viner R, Allen E, Bonell C (2021). Moderated mediation analyses to assess intervention mechanisms for impacts on victimisation, psycho-social problems and mental wellbeing: evidence from the INCLUSIVE realist randomized trial. Soc Sci Med..

